# The effect of cynaropicrin, a sesquiterpene lactone, on the migratory properties of triple-negative breast cancer cells and the underlying mechanisms

**DOI:** 10.22038/ajp.2025.25894

**Published:** 2026

**Authors:** Meriana Barreto Amaral, Hamad Ali Hamad, Soumya V Menon, Mandeep Kaur, Gv Sivaprasad, Wesam R. Kadhum, Subasini Uthirapathy, Muhammad Ikram Ullah, Mohammed Abed Jawad, Yasser Fakri Mustafa

**Affiliations:** 1 *Master of midwifery Researcher and Lecturer Universidade Nacional Timor Lorosae, Dili, Timor Lorosae*; 2 *Department of pathological analysis, collage of applied sciences, University of Fallujah Al-Anbar, Iraq*; 3 *Department of Chemistry and Biochemistry, School of Sciences, JAIN (Deemed to be University), Bangalore, Karnataka, India*; 4 *Department of Sciences, Vivekananda Global University, Jaipur, Rajasthan-303012, India*; 5 *Department of Basic Science & Humanities, Raghu Engineering College, Visakhapatnam, India*; 6 *Department of Pharmacy, Kut University College, Kut 52001, Wasit, Iraq*; 7 *Advanced Research Center, Kut University College, Kut 52001, Wasit, Iraq*; 8 *Pharmacy Department, Tishk International University, Erbil, Kurdistan Region, Iraq*; 9 *Department of Clinical Laboratory Sciences, College of Applied Medical Sciences, Jouf University, Sakaka-72388, Aljouf, Saudi Arabia*; 10 *Department of Pharmaceutics, Al-Nisour University College, Baghdad/Iraq*; 11 *Department of Pharmaceutical Chemistry, College of Pharmacy, University of Mosul, Mosul-41001, Iraq*

**Keywords:** Triple-negative breast cancer Migration, Epithelial-mesenchymal transition, Angiogenesis, Cynaropicrin

## Abstract

**Objective:**

Triple-negative breast cancer (TNBC) is the most metastatic type of breast cancer. Cynaropicrin, a sesquiterpene lactone, shows potential anticancer effects. This study evaluated cynaropicrin's impact on metastasis and angiogenesis in TNBC cells.

**Materials and Methods:**

MDA-MB-231 and MDA-MB-468 cell lines were exposed to incrementing concentrations of cynaropicrin. The proliferation of the cell lines was assayed using the MTT method. A wound scratch technique was chosen to appraise the migratory properties of cells following cynaropicrin treatment. The transcript levels of epithelial-mesenchymal transition (EMT) and pro-angiogenic factors were quantified via quantitative polymerase chain reaction. The western blotting technique estimated the amount of E-cadherin, N-cadherin, Fibronectin, Vimentin, and VEGFA.

**Results:**

The proliferation of MDA-MB-231 and MDA-MB-468 cells was significantly lowered due to cynaropicrin in a concentration-associated way. Results of the wound healing method uncovered that cynaropicrin could mitigate the migration of breast-derived MDA-MB-231 and MDA-MB-468 cells. Cynaropicrin also upregulated E-cadherin and hindered the protein expression of N-cadherin, Vimentin, Fibronectin 1, and VEGFA in breast-derived MDA-MB-468 and MDA-MB-231 cells.

**Conclusion:**

The present findings indicated the anti-metastatic capacity of cynaropicrin against TNBC by a mechanism that implicated the inhibition of the EMT and pro-angiogenic factor VEGFA. These outcomes suggest cynaropicrin as an anti-metastatic and anti-angiogenic sesquiterpene lactone against TNBC.

## Introduction

Breast cancer is the most prevalent type of cancer throughout the globe (Wilkinson and Gathani 2022). According to recent estimates, patients with breast tumors may augment to more than 3 million cases with a mortality rate of 1 million per year by 2040 (Arnold et al. 2022). Among a variety of breast cancers, triple-negative breast cancer (TNBC) is a particular histologic type with highly aggressive features, poor clinical outcomes, high aggressive metastatic capability, high recurrence, and poor prognosis (Medina et al. 2020). TNBC tumors generally show resistance to therapeutic agents due to the loss of three important receptors, including estrogen receptor (ER), progesterone receptor (PR), or human epidermal growth factor receptor 2 (HER2) (Kamalabadi-Farahani et al. 2025; Yin et al. 2020). Classical methods to cure breast tumor patients are surgery, hormone therapy, radiotherapy, and chemotherapy. Patients with TNBC tumors are recommended to be treated with chemotherapeutic agents to enhance therapy of the disease due to the absence of necessary receptors (Kumar and Aggarwal 2016). However, breast surgery with radiotherapy or without it, or breast keeping with radiotherapy is another well-established therapy for TNBC patients (Kumar and Aggarwal 2016). Recently, several specific targeted therapies have been created for the suppression of different types of breast tumors (Masoud and Pagès 2017). The Food and Drug Administration (FDA) has verified three molecular targeted therapeutics that include olaparib and talazoparib for patients bearing a germline breast cancer gene (*BRCA*) mutation and combination of atezolizumab and nab-paclitaxel for subjects who are positive for programmed death-ligand 1 (*PD-L1+*) (Lyons 2019). 

Metastasis is a process with several stages that is initiated by the invasion of the metastatic cancer cell into the adjacent tissue followed by several steps such as intravasation, circulation in the blood or lymph, extravasation, and colonization within the distant organ (Hosseini et al. 2015; Rajabi et al. 2023). The first phase in the course of metastasis is indicated by the epithelial-mesenchymal transition (EMT), which is a process by which the cancer cell obtains metastatic characteristics (Hajimehdipoor et al. 2023). Angiogenesis is another important mechanism by which the tumors can easily metastasize into distant organs (Rajabi et al. 2019). This process is a fundamental step in the promotion and advancement of metastasis. 

Sesquiterpene lactones are sesquiterpenoids known for their diverse pharmacological properties (Forouzanfar et al. 2020; Noori et al. 2014; Rajabi et al. 2024). Cynaropicrin, as a sesquiterpene lactone derived from *Cynara cardunculus,* has several biological activities against parasites, aging, hyperlipidemia, inflammation, and cancer (Elsebai et al. 2016; Fuhr et al. 2022). Its anti-cancer effects have been numerously mentioned in the literature (Choi et al. 2005; Muhammad et al. 2003; Yang et al. 2008). However, no previous investigation has studied the influence of cynaropicrin on invasion, migration, and metastasis in TNBC cells. Thus, this current investigation was conducted for assessing the anti-metastatic and anti-angiogenic impacts of cynaropicrin on TNBC cell lines, including MDA-MB-231 and MDA-MB-468, and underlying mechanisms.

## Materials and Methods

### Cell culture and materials

Human TNBC cell lines, MDA-MB-468 and MDA-MB-231, were bought from Sigma Company, USA. The TNBC cell lines were cultured in Dulbecco's Modified Eagle Medium (DMEM) medium at 37°C with 98% humidity and 5% CO_2_ in a standard incubator. Fetal bovine serum, penicillin/streptomycin, and trypsin for cell culture were sourced from Gibco Co., Germany. Cynaropicrin was obtained from Sigma Company, USA. The solvent for the preparation of cynaropicrin solution to treat the cells was dimethyl sulfoxide (DMSO; Merck, Germany).

### Cell viability

Both MDA-MB-231 and MDA-MB-468 cells were cultivated at an amount of 10^4^ cells in each well in 96-well cell culture plates. Afterward, both cell lines received augmenting concentrations of cynaropicrin (2, 4, 6, 8, 10, 15, 20, and 30 μM) for 24, 48, and 72 hr. DMEM was administered to the control wells with sham wells receiving DMSO solution. Subsequently, the MTT viability assay was performed to estimate the proliferation percent of the cells. Briefly, the MTT reagent was applied at an amount of 5 mg/ml followed by the incubation with both cancer cell lines for 4 hr. Then, the MTT solution was discarded and 200 µl of DMSO solution was poured into each well. Finally, the formazan solution’s absorbance was read at 570 and 630 nm using a plate reader spectrophotometer (Epoch, USA).

### Wound scratch technique

Using *in-vitro* wound scratch technique, the possible influence of cynaropicrin treatment on the invasion and migration of MDA-MB-468 and MDA-MB-231 breast-derived cell lines was examined. Briefly, two TNBC cell lines were cultivated in 6-well cell culture plates at an amount of 2 × 10^5^ cells per well. The cultured cell lines were permitted to stick and proliferate as 50–60%. Then, three horizontal scratches were made in each well using 200 µl yellow pipette tips. To remove cell debris, washing of each well was done twice using Phosphate Buffered saline (PBS) buffer. Subsequently, the treatment wells containing MDA-MB-231 cells received cynaropicrin at doses of 4 (IC25) and 8 (IC50) μM and the wells that contained MDA-MB-468 cells received 5 (IC25) and 10 (IC50) μM of cynaropicrin. The control wells were administered with a standard DMEM culture medium. Afterward, the pictures of all cynaropicrin-treated and control wells were grabbed at 0, 12, 24, 48, and 72 hr. 

### Gene transcript expression examination

The amount of gene markers related to EMT (Cadherin 1: *CDH1*, Cadherin 2: *CDH2*, Vimentin: *VIM*, and fibronectin 1: *FN1*) and angiogenic marker Vascular Endothelial Growth Factor A (*VEGFA*) in MDA-MB-468 and MDA-MB-231 breast-derived cell lines was measured using quantitative real-time PCR method. 

RNA molecules were isolated by utilizing an RNeasy mini kit (Qiagen, Hilden, Germany) following the protocols of the manufacturer. RNA of samples was reversed to cDNA by utilizing a standard kit for cDNA synthesis (Qiagen, Hilden, Germany). Amplification of cDNA molecules was carried out by SYBR-green master mix (Ampliqon, Denmark) on ABI PRISM 7900HT (Applied Biosystems, USA). Melting curves were analyzed for each product to confirm the specificity of primers and products. The expression level of the transcripts of the *GAPDH* gene was chosen as the housekeeping gene and the expression of all transcripts was estimated by using the 2^-ΔΔCt^ method. Table 1 lists the primer sequences used in this study for gene amplification.

**Table 1:** Primer sequences used in the present study

**Gene name**	**Forward primer**	**Reverse primer**
*CDH1*	5'-GGGGTCTGTCATGGAAGGTG-3′	5'-CGACGTTAGCCTCGTTCTCA-3′
*CDH2*	5'-GCGTCTGTAGAGGCTTCTGG-3′	5'-GCCACTTGCCACTTTTCCTG-3′
*FN1*	5'-ACAAGCATGTCTCTCTGCCAA-3′	5'-TCAGGAAACTCCCAGGGTGA-3′
*VIM*	5'-TCCGCACATTCGAGCAAAGA-3′	5'-ATTCAAGTCTCAGCGGGCTC-3′
*VEGFA*	5'-GAGCAAGACAAGAAAATCCC-3′	5'-CCTCGGCTTGTCACATCTG-3′
*GAPDH*	5'-ACCCACTCCTCCACCTTTGA-3′	5'-CT GTTGCTGTAGCCAAATTCGT-3′

### Protein expression analysis

The influence of cynaropicrin treatment on the amount of EMT protein concentrations, which include E-cadherin, N-cadherin, Vimentin, and Fibronectin 1 together with an angiogenic factor VEGFA, was estimated in MDA-MB-468 and MDA-MB-231 breast-derived cell lines by utilizing western blotting. Briefly, the cells were trypsinized and subjected to centrifugation (2500 RPM) for 7 min. Then, both cell lines were washed with PBS and incubated with radioimmunoprecipitation assay buffer (RIPA) lysis buffer (Thomas Scientific Inc., USA). RIPA buffer lysed the harvested cancer cells, and the resulting lysate was centrifuged. The supernatants underwent western blotting, with total protein content measured by the Bradford method. Next, 40 µg of total protein was mounted onto 10% SDS-PAGE and transferred to a standard PVDF membrane. Upon the blockage of the membrane by utilizing 5% non-fat milk, primary antibodies namely E-cadherin, N-cadherin, Vimentin, Fibronectin 1, VEGFA, and GAPDH (Santa Cruz Biotech, Santa Cruz, USA) were added onto the membrane. The membrane was incubated overnight at 4˚C, followed by the addition of horseradish peroxidase (HRP)–conjugated secondary antibody for 1 hour in the dark. Finally, the immunoblots were observable by applying an enhanced chemiluminescent standard kit (Bio-Rad, CA, USA) and exposed to autoradiography film.

### Statistical assessments

Data were analyzed using one-way ANOVA followed by Tukey's *post hoc* test. Results from triplicate experiments are presented as mean ± standard deviation (SD). Statistical analyses were conducted with GraphPad PRISM software version 8, and p-values less than 0.05 were deemed statistically significant.

## Results

### Impact of cynaropicrin compound on the viability rates of MDA-MB-231 cell line

**Figure 1:**
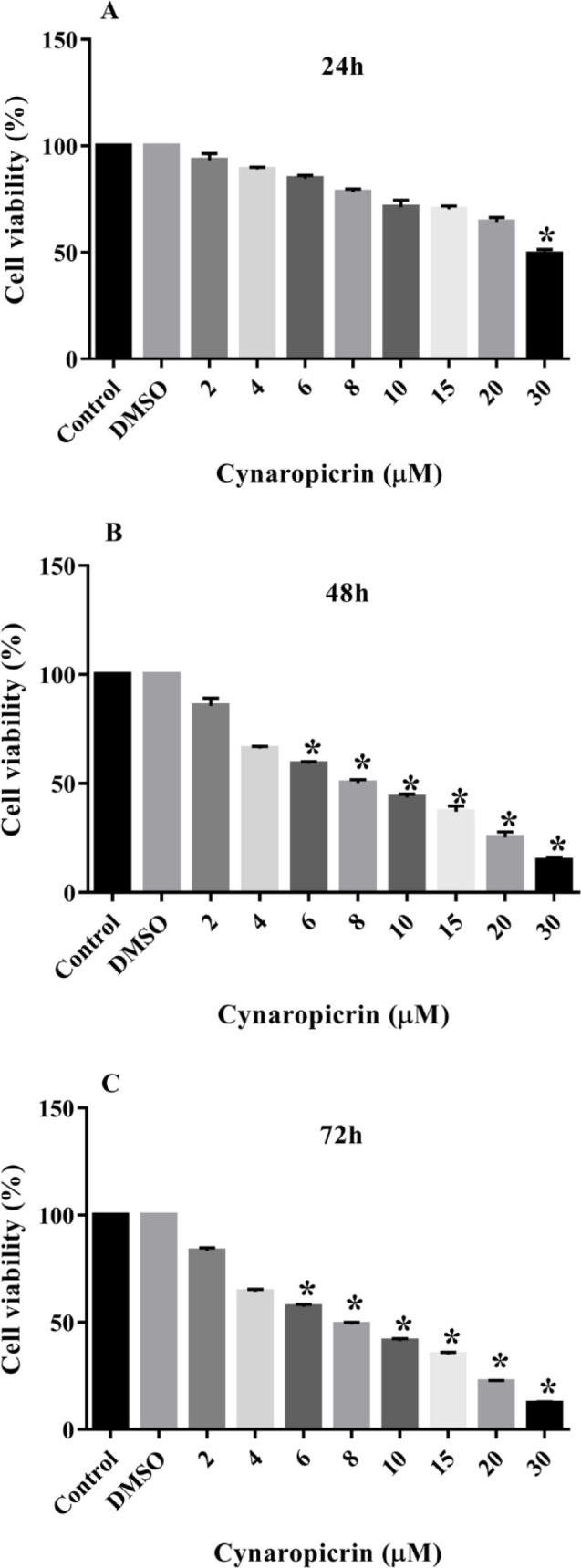
Anti-proliferative effect of cynaropicrin on the MDA-MB-231 cell line. Different concentrations of cynaropicrin (2, 4, 6, 8, 10, 15, 20, and 30 µM) were used to treat MDA-MB-231 cells for 24 (A), 48 (B), and 72 (C) hr. The MTT method was used to measure the viability of the cells. The data are expressed as mean ± SD of three independent experiments. *p<0.05 as compared to the control was considered statistically significant.

Cynaropicrin treatment for 24 hr declined the proliferation of the MDA-MB-231 cells at doses that are greater than 30 µM but lower concentrations had no significant effect on their proliferation rate (p=0.063) (Figure 1A). Treatment of these cells for 48 hr significantly inhibited their proliferation at doses that are greater than 6 µM with the inhibitory concentration 50 (IC50) value of 8.05 µM (p=0.04) (Figure 1B). Cynaropicrin treatment for 72 hr also suppressed MDA-MB-231 cell proliferation at doses that are greater than 6 µM (p=0.032). The IC50 value of cynaropicrin hindering impact on these cancer cells for 72 hr was 7.96 µM (Figure 1C).

### Impact of Cynaropicrin compound on the viability rates of MDA-MB-468 cell line

**Figure 2:**
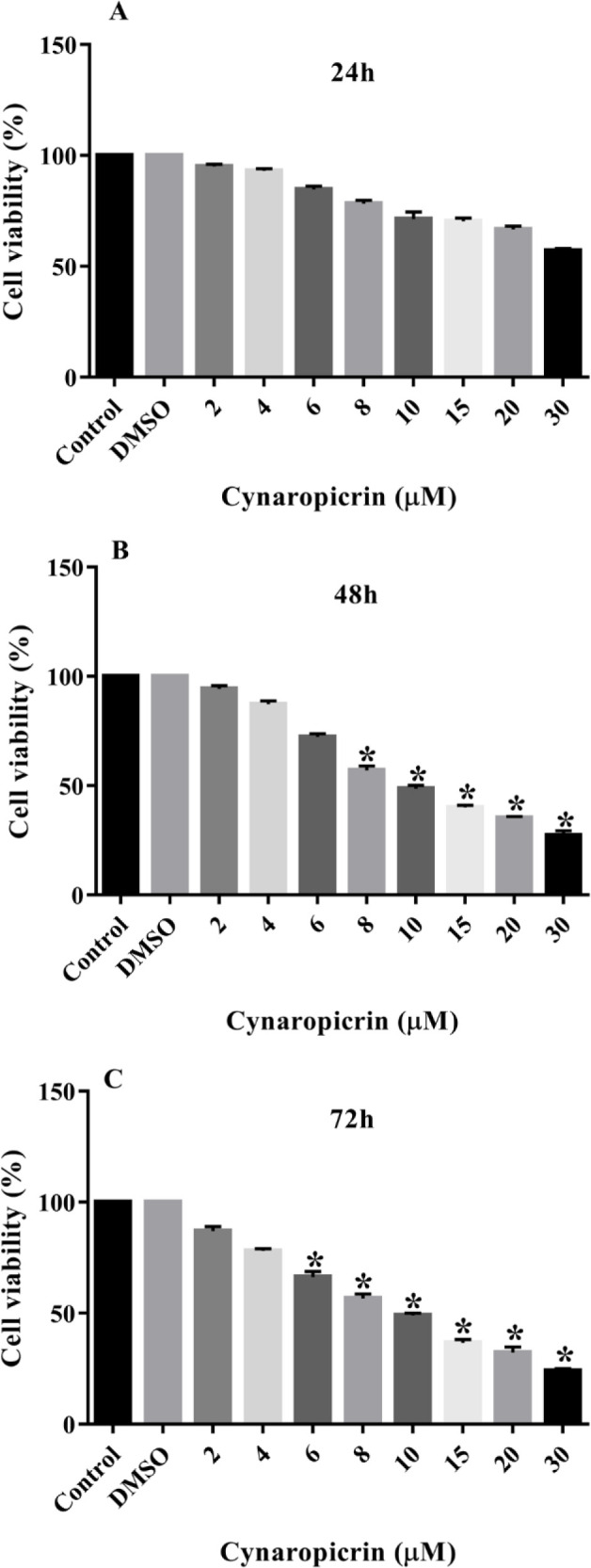
Anti-proliferative effects of cynaropicrin on the MDA-MB-468 cell line. The cells were treated with different doses of cynaropicrin (2, 4, 6, 8, 10, 15, 20, and 30 µM) for 24 (A), 48 (B), and 72 (C) hr. The cell viability was determined using MTT assay. The data are expressed as mean ± SD of three independent experiments. *p<0.05 as compared to the control was considered statistically significant.

Treatment of the MDA-MB-468 cell line with incrementing doses of cynaropicrin (2-30 µM) for 24, 48, and 72 hr is illustrated in Figures 2A, B, and C. The obtained data suggested that cynaropicrin prohibiting impact on the proliferation of these breast tumor cells in a dose-associated way. In detail, cynaropicrin administration to the MDA-MB-468 cell line for 24 hr had no marked effects on their proliferation (p=0.07) (Figure 2A). However, exposing of these cancer cells to augmenting doses of cynaropicrin for 48 (p=0.042) and 72 hr (p=0.04) markedly hindered their proliferation with IC50 values of 9.90 and 9.95, respectively.

### Effects of cynaropicrin on the propagation of MDA-MB-231 and MDA-MB-468 cell lines

**Figure 3:**
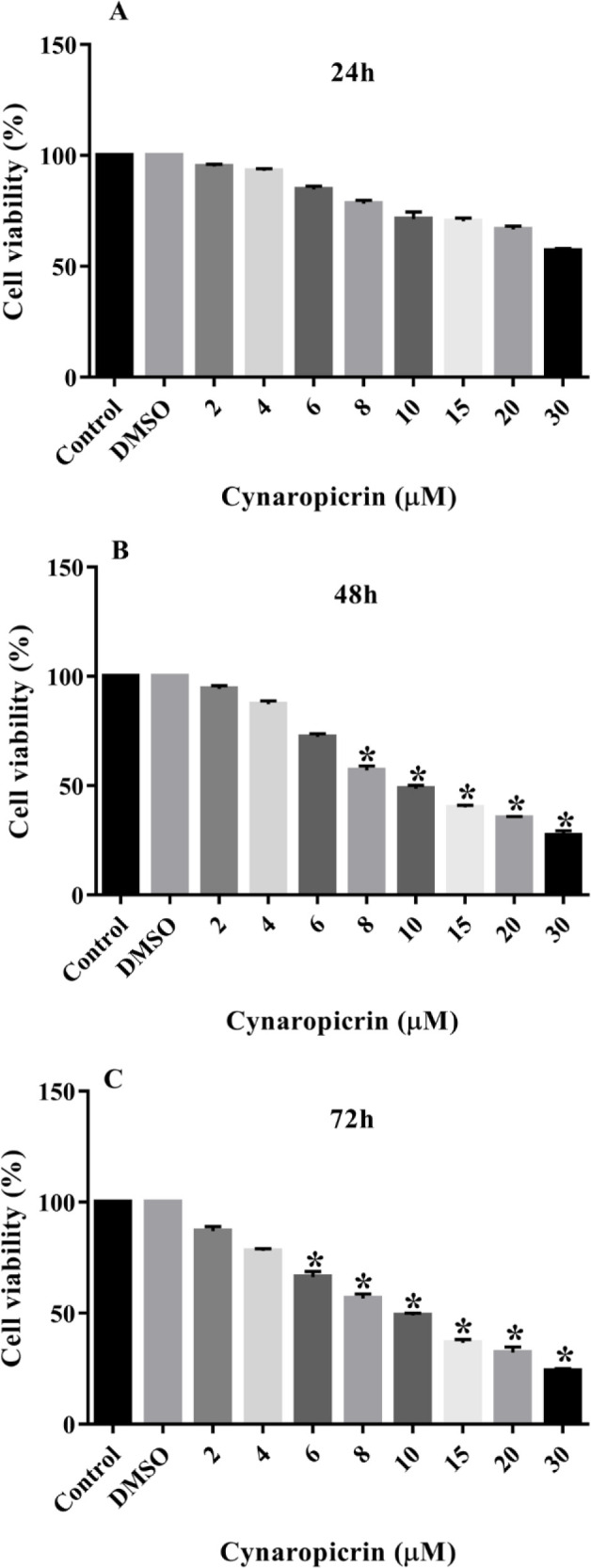
(A) Anti-invasion effect of cynaropicrin on MDA-MB-231 cell line at the concentrations of 4 (IC_25_) and 8 (IC_50_) µM for 48 hr. (B) Anti-invasion effect of cynaropicrin on MDA-MB-468 cell line at the concentrations of 5 (IC_25_) and 10 (IC_50_) µM for 48 hr. The scratch assay was performed in triplicate with three horizontal scratches within each well.

To examine the influence of cynaropicrin on the invasiveness and migratory capacity of MDA-MB-231 and MDA-MB-468 cell lines, these malignant cell lines were exposed to IC25 and IC50 concentrations of this phytochemical drug and anti-migration activity of the compound was evaluated using wound healing assay. The outcomes revealed that cynaropicrin at a concentration of 8 μM (IC50) considerably inhibited the migration of the MDA-MB-231 cell line even after 48 hr in comparison to untreated control cells (Figure 3A). However, treating MDA-MB-231 cells with cynaropicrin at a concentration of 4 μM (IC25) elicited no marked impact on the migratory properties of these cells compared to the untreated cells. Figure 3B indicates that cynaropicrin treatment prohibited the invasion and migration of MDA-MB-468 cells at all amounts of this natural material. However, the cells treated with IC50 concentration of cynaropicrin more considerably hampered the invasiveness of these TNBC cells in comparison to the cells treated with IC25 concentration of the drug.

### Influence of cynaropicrin on gene expression in MDA-MB-231 and MDA-MB-468 cell lines

**Figure 4:**
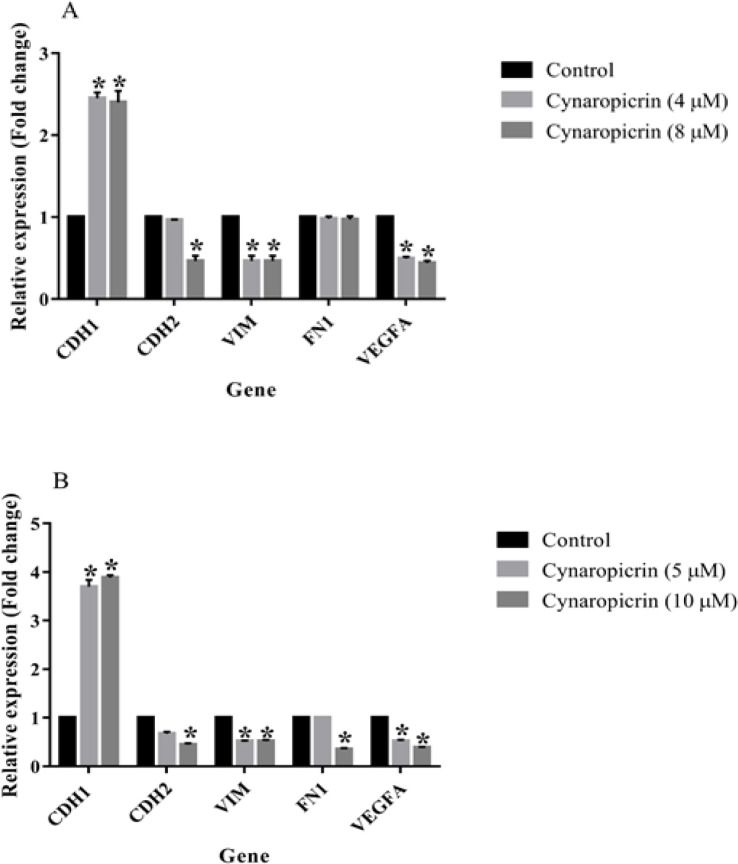
. (A) Effect of cynaropicrin treatment at the concentrations of 4 (IC_25_) and 10 (IC_50_) µM for 48 hr on the expression of CDH1, CDH2, VIM, FN1, and VEGFA genes in the MDA-MB-231 cell line. (B) Effect of cynaropicrin ate doses of 5 (IC_25_) and 10 (IC_50_) µM for 48 hr on the mRNA expression of CDH1, CDH2, VIM, FN1, and VEGFA genes in the MDA-MB-468 cell line. The experiments were performed in triplicate. The results are representative of means ± SD. *p<0.05 as compared to the control was considered statistically significant.

The effects of cynaropicrin use (48 hr) on the gene expression of EMT markers such as *CDH1*, *CDH2*, *VIM*, and *FN1* along with the angiogenesis activator gene *VEGFA* were measured both in MDA-MB-468 and MDA-MB-231 breast-derived cell lines. According to the data shown in Figure 4A, cynaropicrin, at amounts of 4 and 8 µM, meaningfully increased the levels of the *CDH1* gene (p=0.046) along with no influence on the levels of the *FN1* gene (p=0.08) in the MDA-MB-231 cell line compared with the control. It also downregulated the mRNA expression of *CDH2* (p=0.045) at the amount of 4 µM and lowered the levels of *VIM* (p=0.038) and *VEGFA* (p=0.043) genes at doses of 4 and 8 µM in MDA-MB-231 cells. In the MDA-MB-468 cell line, cynaropicrin treatment (5 and 10 µM) markedly elevated the transcript level of the *CDH1* (p=0.031) gene and decreased the expression levels of *VIM* (p=0.037) and *VEGFA* (p=0.033) genes when comparing the control cells (Figure 4B). This sesquiterpene lactone also hindered the expression of *CDH2* (p=0.032) and *FN1* (p=0.04) mRNA levels at a dose of 5 µM in the MDA-MB-468 cells when comparing with the control cells.

### Effects of cynaropicrin on protein expression in MDA-MB-231 and MDA-MB-468 cell lines

**Figure 5:**
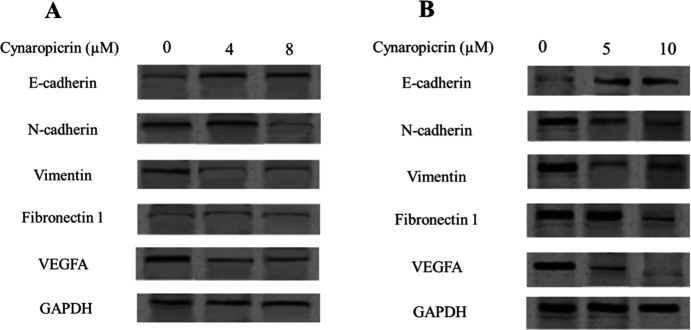
. (A) Effect of cynaropicrin treatment on the expression of E-cadherin, N-cadherin, vimentin, fibronectin 1, and VEGFA at protein levels in the MDA-MB-231 cell line. (B) Effect of cynaropicrin on the protein expression of E-cadherin, N-cadherin, vimentin, fibronectin 1, and VEGFA in the MDA-MB-468 cell line. The experiments were performed in triplicate.

The protein expression of EMT and angiogenic markers in cynaropicrin-treated MDA-MB-231 and MDA-MB-468 cell lines was measured using western blotting. The results of western blotting illustrated that treatment of MDA-MB-231 cells with cynaropicrin ( 4 and 8 µM for 48 hr) markedly heightened the protein expression of E-cadherin and lowered VEGFA and vimentin compared with untreated MDA-MB-231 cells (Figure 5A). Cynaropicrin, at a dose of 8 µM, diminished the concentration of N-cadherin protein in these cells. However, no remarkable effect was shown in the amount of fibronectin 1 protein in cynaropicrin-treated MDA-MB-231 cells. As depicted in Figure 5B, the protein expression of E-cadherin was increased and the protein level of N-cadherin, vimentin, and VEGFA was decreased in MDA-MB-468 cells treated with cynaropicrin (5 and 10 µM) as compared with the control. In addition, cynaropicrin at a dose of 10 µM reduced the amount of fibronectin 1 protein in MDA-MB-468 cells in comparison to untreated cells.

## Discussion

In this current investigation, the results of the MTT technique uncovered that cynaropicrin treatment for 48 and 72 hr diminished the augmentation of the MDA-MB-231 cells with the IC_50_ values of 8.05 µM and 7.96 µM, respectively. Also, treatment of MDA-MB-468 cells with incrementing amounts of cynaropicrin for 48 and 72 hr meaningfully blocked their proliferation with IC_50_ values of 9.90 µM and 9.95 µM, respectively. Accordingly, the other experiments in the present study were performed by treating MDA-MB-468 and MDA-MB-231 cells with IC_50_ and IC_25_ concentrations of cynaropicrin after 48 hr treatment period. In the second step, a wound scratch assay was performed to assess the influence of cynaropicrin on the invasive and migratory capability of MDA-MB-231 and MDA-MB-468 cell lines. The outcomes of scratch wound assay on MDA-MB-231 unraveled that cynaropicrin (8 μM) markedly reduced the migration of the MDA-MB-231 cell line. Moreover, cynaropicrin treatment (5 and 10 μM) inhibited the invasion and migration of MDA-MB-468 cells. In the final step, the effect of cynaropicrin on the amount of EMT indexes (E-cadherin, N-cadherin, Vimentin, and Fibronectin 1) and angiogenic factor VEGFA was investigated at mRNA and protein levels to uncover the mechanism of anti-metastatic activity of cynaropicrin in both TNBC cell lines. Looking at the results of western blotting technique and real-time PCR, cynaropicrin-treated MDA-MB-468 and MDA-MB-231 cells showed augmented expression of E-cadherin and declined levels of N-cadherin, Vimentin, Fibronectin 1, and VEGFA when compared with untreated control cells.

The anticancer activity of cynaropicrin has been proven in several previous studies in the literature (Boulos et al. 2023; Lepore et al. 2019; Li et al. 2021; Yang et al. 2022). In a study on the *in-vitro* and *in-vivo* models of colorectal cancer (CRC), cynaropicrin suppressed the proliferation of CRC cells and initiated apoptosis by a mechanism that induced the activation of the JAK/STAT signaling pathway (Zheng et al. 2020a). In another study on a human CRC cell line, HCT-116, cynaropicrin administration considerably triggered apoptosis in these cancer cells by acting on the intrinsic pathway of apoptosis (Wang et al. 2023). Rotondo et al. unraveled that cynaropicrin-exposed human glioblastoma cell line, U-87MG, underwent cell death in a dose-associated manner. They also unraveled that apoptosis was the prominent cell death mechanism by which this cancer cell line died following cynaropicrin treatment (Rotondo et al. 2022). To the best of our knowledge, only three studies have evaluated the anti-metastatic or anti-invasion properties of cynaropicrin on cancer cells. For example, De Cicco et al. investigated to examine pro-apoptotic and anti-metastatic roles of cynaropicrin in human melanoma cell line A375. They indicated that cynaropicrin significantly hampered the proliferation and augmented apoptosis of A375 cells. It also prohibited the migration, invasion, and colony formation of these cancer cells by blocking mitogen-activated protein kinase (MAPK) and nuclear factor kappa B (NF-κB) pathways (De Cicco et al. 2021). In another study, the migration potential of three human CRC cell lines was estimated using a transwell assay. The results of that study unraveled that cynaropicrin markedly prevented the cell migration of CRC cells compared with the control group (Zheng et al. 2020b). The outcomes of the third study suggested that cynaropicrin may act as an anti-metastasis agent in leukocyte cancer cells (Cho et al. 2004). 

To date, no prior investigation has explored the anti-metastatic capability of cynaropicrin in breast cancer cells. Therefore, the current research is the first report of the anti-metastasis function of cynaropicrin in TNBC cells as the most invasive and highly metastatic subtype of breast cancer. Our findings provided in-vitro evidence to highlight the inhibitory effect of this sesquiterpene lactone on the invasion, migration, or metastatic propagation of two distinct TNBC cell lines. The present data also suggested a potential mechanism by which cynaropicrin hindered the migratory activity of TNBC cells. This mechanism may acted by the blockage of the EMT process. This process is the first step in the promotion of metastasis by which the cancer cells gain the ability to invade the adjacent tissue (Rajabi et al. 2020; Ribatti et al. 2020). Metastatic cancer cells undergo the EMT process by downregulating E-cadherin and upregulating N-cadherin, Vimentin, and Fibronectin 1 to obtain mesenchymal characteristics for migration to distant organs (Francou and Anderson 2020). Angiogenesis is another critical process that is pivotal for the metastatic propagation of cancer cells (Liu et al. 2023). In addition, angiogenesis provides oxygen, nutrients, and growth factors for growing tumors (Al-Ostoot et al. 2021). The process of angiogenesis is a dynamic pathway modulated by different pro- and anti-angiogenic factors (Larionova et al. 2021). Vascular endothelial growth factor (VEGF) family members are key inducers of angiogenesis for the stimulation of endothelial cells for neovascularization (Melincovici et al. 2018). Decreased expression of VEGFA in the present investigation may underscore the anti-angiogenic and anti-metastatic activity of cynaropicrin against TNBC invasiveness. 

The strengths of the present investigation is the simultaneous study of the influence of phytochemical cynaropicrin on two types of TNBC cell lines with some different features. The other strength of the current study is uncovering the probable mechanism of cynaropicrin anti-metastatic action on TNBC cells. The weakness of the study may arise from budget limitations that inhibited a comprehensive examination of some other key metastatic factors. Due to some budget limitations, we could not assess extracellular matrix factors associated with metastatic cascade of breast cancer cells. Therefore, exaltation of these factors might be incorporated into future work.

The present study provided evidence to unravel the anti-metastatic or anti-migratory influences of cynaropicrin on two different TNBC cell lines MDA-MB-231 and MDA-MB-468. Our data also highlighted a novel mechanism by which cynaropicrin hindered the migration and invasion of these TNBC cells. This mechanism involved the blockage of the EMT process to hamper the migratory properties of both TNBC cell lines. Further experiments showed that cynaropicrin downregulated the levels of VEGFA as a key inducer of angiogenesis, which is pivotal for cancer cell metastasis. This study is the first to evaluate the anti-metastatic activity of cynaropicrin in breast cancer cells, specifically in TNBC, which is known for its invasiveness and high metastatic potential. Our in vitro results demonstrate that this sesquiterpene lactone effectively inhibits invasion, migration, and metastatic spread in two different TNBC cell lines. Taken together, the present results may propose cynaropicrin as a novel and effective drug for blocking metastasis in TNBC.

## References

[1] Al-Ostoot FH, Salah S, Khamees HA, Khanum SA (2021). Tumor angiogenesis: Current challenges and therapeutic opportunities. Cancer Treat Res Communi..

[2] Arnold M, Morgan E, Rumgay H, Mafra A, Singh D, Laversanne M (2022). Current and future burden of breast cancer: Global statistics for 2020 and 2040. Breast..

[3] Boulos JC, Omer EA, Rigano D, Formisano C, Chatterjee M, Leich E (2023). Cynaropicrin disrupts tubulin and c-Myc-related signaling and induces parthanatos-type cell death in multiple myeloma. Acta Pharmacol Sin.

[4] Cho JY, Kim AR, Joo HG, Kim BH, Rhee MH, Yoo ES (2004). Cynaropicrin, a sesquiterpene lactone, as a new strong regulator of CD29 and CD98 functions. Biochem Biophys Res Communi.

[5] Choi SZ, Choi SU, Lee KR (2005). Cytotoxic sesquiterpene lactones from Saussurea calcicola. Arch Pharm Res.

[6] De Cicco P, Busà R, Ercolano G, Formisano C, Allegra M, Taglialatela-Scafati O (2021). Inhibitory effects of cynaropicrin on human melanoma progression by targeting MAPK, NF-κB, and Nrf-2 signaling pathways in vitro. Phytothera Res.

[7] Elsebai MF, Mocan A, Atanasov AG (2016). Cynaropicrin: A Comprehensive Research Review and Therapeutic Potential As an Anti-Hepatitis C Virus Agent. Front Pharmacol..

[8] Forouzanfar F, Ghazavi H, Vahedi MM, Tarrah K, Yavari Z, Hosseini A (2020). Tanacetum parthenium enhances pentobarbital-induced sleeping behaviors. Avicenna J Phytome.

[9] Francou A, Anderson KV (2020). The Epithelial-to-Mesenchymal Transition (EMT) in Development and Cancer. Annu Rev Cancer Bio..

[10] Fuhr L, Basti A, Brás TS, Duarte MF, Relógio A (2022). Antiproliferative Effects of Cynara Cardunculus in Colorectal Cancer Cells Are Modulated by the Circadian Clock. Int J Mol Sci.

[11] Hajimehdipoor H, Tahmasvand Z, Nejad FG, Maresca M, Rajabi S (2023). Rutin Promotes Proliferation and Orchestrates Epithelial-Mesenchymal Transition and Angiogenesis in MCF-7 and MDA-MB-231 Breast Cancer Cells. Nutrients..

[12] Hosseini A, Mousavi SH, Ghanbari A, Homaee Shandiz F, Raziee HR, Pezeshki Rad M (2015). Effect of saffron on liver metastases in patients suffering from cancers with liver metastases: A randomized, double blind, placebo-controlled clinical trial. Avicenna J Phytomed.

[13] Kamalabadi-Farahani M, Jamshidi Adegani F, Karimi R, Atashi A (2025). Anti-cancer effects of frankincense methanolic extract on brain metastatic breast cancer cells. Avicenna J Phytomed,.

[14] Kumar P, Aggarwal R (2016). An overview of triple-negative breast cancer. Arch Gynecol Obstet.

[15] Larionova I, Kazakova E, Gerashchenko T, Kzhyshkowska J (2021). New Angiogenic Regulators Produced by TAMs: Perspective for Targeting Tumor Angiogenesis. Cancers (Basel).

[16] Lepore SM, Maggisano V, Lombardo GE, Maiuolo J, Mollace V, Bulotta S (2019). Antiproliferative Effects of Cynaropicrin on Anaplastic Thyroid Cancer Cells. Endocr Metab Immune Disord Drug Targets.

[17] Li W, Xu X, Wan Y, Wang H, Tao H, Huang H (2021). Cynaropicrin inhibits lung cancer proliferation by targeting EGFR/AKT signaling pathway. Trop J Pharmaceut Res.

[18] Liu ZL, Chen HH, Zheng LL, Sun LP, Shi L (2023). Angiogenic signaling pathways and anti-angiogenic therapy for cancer. Signal Transduct Target Therap.

[19] Lyons TG (2019). Targeted Therapies for Triple-Negative Breast Cancer. Curr Treat Options Oncol.

[20] Masoud V, Pagès G (2017). Targeted therapies in breast cancer: New challenges to fight against resistance. World J Clin Oncol.

[21] Medina MA, Oza G, Sharma A, Arriaga LG, Hernández Hernández JM, Rotello VM (2020). Triple-Negative Breast Cancer: A Review of Conventional and Advanced Therapeutic Strategies. Int J Environ Res Public Health.

[22] Melincovici CS, Boşca AB, Şuşman S, Mărginean M, Mihu C, Istrate M (2018). Vascular endothelial growth factor (VEGF) - key factor in normal and pathological angiogenesis. Rom J Morphol Embryo.

[23] Muhammad I, Takamatsu S, Mossa JS, El-Feraly FS, Walker LA, Clark AM (2003). Cytotoxic sesquiterpene lactones from Centaurothamnus maximus and Vicoa pentanema. Phytothera Res.

[24] Noori A, Amjad L, Yazdani F (2014). The effects of Artemisia deserti ethanolic extract on pathology and function of rat kidney. Avicenna J Phytomed.

[25] Rajabi S, Dehghan MH, Dastmalchi R, Jalali Mashayekhi F, Salami S, Hedayati M (2019). The roles and role-players in thyroid cancer angiogenesis. Endocr J.

[26] Rajabi S, Irani M, Moeinifard M, Hamzeloo-Moghadam M (2024). Britannin suppresses MCF-7 breast cancer cell growth by inducing apoptosis and inhibiting autophagy. Avicenna J Phytomed.

[27] Rajabi S, Rajani HF, Mohammadkhani N, Ramírez-Coronel AA, Maleki M, Maresca M (2023). Long Non-Coding RNAs as Novel Targets for Phytochemicals to Cease Cancer Metastasis. Molecules..

[28] Rajabi S, Shakib H, Dastmalchi R, Danesh-Afrooz A, Karima S, Hedayati M (2020). Metastatic propagation of thyroid cancer; organ tropism and major modulators. Future Onco.

[29] Ribatti D, Tamma R, Annese T (2020). Epithelial-Mesenchymal Transition in Cancer: A Historical Overview. Transl Onco.

[30] Rotondo R, Oliva MA, Arcella A (2022). The Sesquiterpene Lactone Cynaropicrin Manifests Strong Cytotoxicity in Glioblastoma Cells U-87 MG by Induction of Oxidative Stress. Biomedicines.

[31] Wang L, Bie X, Mickymaray S, Alothaim AS, Pei Y, Gong H (2023). Induction of Apoptosis by Cynaropicrin in Human Colon Cancer Cell Line HCT-116 through the Mitochondria-mediated Apoptotic Pathway. Pharmacogn Magaz.

[32] Wilkinson L, Gathani T (2022). Understanding breast cancer as a global health concern. Br J Radiol.

[33] Yang MC, Choi SU, Choi WS, Kim SY, Lee KR (2008). Guaiane sesquiterpene lactones and amino acid-sesquiterpene lactone conjugates from the aerial parts of Saussurea pulchella. J Nat Prod.

[34] Yang R, Ma S, Zhuo R, Xu L, Jia S, Yang P (2022). Suppression of endoplasmic reticulum stress-dependent autophagy enhances cynaropicrin-induced apoptosis via attenuation of the P62/Keap1/Nrf2 pathways in neuroblastoma. Front Pharmacol..

[35] Yin L, Duan JJ, Bian XW, Yu SC (2020). Triple-negative breast cancer molecular subtyping and treatment progress. Breast Cancer Res.

[37] Zheng D, Zhu Y, Shen Y, Xiao S, Yang L, Xiang Y (2020). Cynaropicrin Shows Antitumor Progression Potential in Colorectal Cancer Through Mediation of the LIFR/STATs Axis. Front Cell Dev Biol..

